# Bovine Neutrophil β-Defensin-5 Provides Protection against Multidrug-Resistant *Klebsiella pneumoniae* via Regulating Pulmonary Inflammatory Response and Metabolic Response

**DOI:** 10.3390/ijms251910506

**Published:** 2024-09-29

**Authors:** Shuxin Zhu, Dejia Dai, Han Li, Jingsheng Huang, Weichao Kang, Yunmei Yang, Yawen Zhong, Yifei Xiang, Chengzhi Liu, Jiakang He, Zhengmin Liang

**Affiliations:** 1 College of Animal Science and Technology, Guangxi University, Nanning 530004, China; 2 Guangxi Zhuang Autonomous Region Engineering Research Center of Veterinary Biologics, Nanning 530004, China; 3 Guangxi Key Laboratory of Animal Reproduction, Breeding and Disease Control, Nanning 530004, China

**Keywords:** *Klebsiella pneumoniae*, bovine neutrophil β-defensin-5, inflammatory response, metabolic response

## Abstract

*Klebsiella pneumoniae* (*K. pneumoniae*), a kind of zoonotic bacteria, is among the most common antibiotic-resistant pathogens, and it causes nosocomial infections that pose a threat to public health. In this study, the roles of synthetic bovine neutrophil β-defensin-5 (B5) in regulating inflammatory response and metabolic response against multidrug-resistant *K. pneumoniae* infection in a mouse model were investigated. Mice were administrated intranasally with 20 μg of B5 twice and challenged with *K. pneumoniae* three days after B5 pretreatment. Results showed that B5 failed to directly kill *K. pneumoniae* in vitro, but it provided effective protection against multidrug-resistant *K. pneumoniae* via decreasing the bacterial load in the lungs and spleen, and by alleviating *K. pneumoniae*-induced histopathological damage in the lungs. Furthermore, B5 significantly enhanced the mRNA expression of TNF-α, IL-1β, Cxcl1, Cxcl5, Ccl17, and Ccl22 and obviously enhanced the rapid recruitment of macrophages and dendritic cells in the lungs in the early infection phase, but significantly down-regulated the levels of TNF-α, IL-1β, and IL-17 in the lungs in the later infection phase. Moreover, RNA-seq results showed that *K. pneumoniae* infection activated signaling pathways related to natural killer cell-mediated cytotoxicity, IL-17 signaling pathway, inflammatory response, apoptosis, and necroptosis in the lungs, while B5 inhibited these signaling pathways. Additionally, *K. pneumoniae* challenge led to the suppression of glycerophospholipid metabolism, the phosphotransferase system, the activation of microbial metabolism in diverse environments, and metabolic pathways in the lungs. However, B5 significantly reversed these metabolic responses. Collectively, B5 can effectively regulate the inflammatory response caused by *K. pneumoniae* and offer protection against *K. pneumoniae*. B5 may be applied as an adjuvant to the existing antimicrobial therapy to control multidrug-resistant *K. pneumoniae* infection. Our study highlights the potential of B5 in enhancing pulmonary bacterial clearance and alleviating *K. pneumoniae*-caused inflammatory damage.

## 1. Introduction

Drug resistance is a severe public health problem worldwide. Antimicrobial resistance may be more deadly than cancer, and will cause 10 million deaths each year by 2050 [1]. The inappropriate use of antibiotics leads to the production of multidrug-resistant bacteria, exacerbating inflammatory responses, and disordering gut microbiota [2,3]. According to an early report, carbapenem-resistant *Enterobacterales* account for more than 1100 deaths in the US and cause economic losses of over USD 130 million every year [4]. *Klebsiella pneumoniae* (*K. pneumoniae*, KP) is a kind of zoonotic bacteria and a common cause of antimicrobial-resistant opportunistic infections in hospitalized patients, and *K. pneumoniae* has emerged as a major clinical and public health threat due to the increasing prevalence of healthcare-associated infections caused by its multidrug-resistant strains [5]. *K. pneumoniae* colonizes respiratory and intestinal tract mucosal surfaces of healthy humans and livestock, and often causes pneumonia, urinary tract infections, liver abscesses, and wound infections that can progress to bacteremia in some cases [6]. Neonates, the elderly, some people with inserted medical devices, and the immunocompromised people are prone to *K. pneumoniae* infection [6]. Moreover, the contamination of retail meats and vegetables with antibiotic-resistant *K. pneumoniae* is a cause of extraintestinal infections in humans [7]. Additionally, *K. pneumoniae* also causes bovine mastitis [8]. Notably, asymptomatic gut colonization by multidrug-resistant *K. pneumoniae* leads to gut dysbiosis and increases the severity of lung infection caused by *Pseudomonas aeruginosa* [9]. Therefore, developing new strategies to control *K. pneumoniae* infection is urgently needed.

One of the strategies to eliminate the problem of antibiotic resistance is to use antimicrobial peptides (AMPs). AMPs have both antibacterial and immunomodulatory properties [10,11]. Research on AMPs are attracting increasing attention owing to their broad-spectrum antibacterial activity, lower levels of pathogen resistance, and high cell compatibility [10,11]. They exert immunomodulatory functions via recruiting immune cells and inducing cytokine and chemokine secretion [10]. The cathelicidin-related antimicrobial peptide was required for effective lung mucosal immunity against *K. pneumoniae* [12]. The predicted cathelicidins derived from *Varanus komodoensis*, WAM-1, and A20L exhibited antibacterial and anti-inflammatory activities against carbapenem-resistant *K. pneumoniae* [13,14,15]. Human neutrophil peptide 1 promoted immune sterilization via reducing the virulence of multidrug-resistant *K. pneumoniae* and enhancing macrophage function [16]. The antimicrobial peptide hLF1-11 from human lactoferrin could synergistically inhibit multidrug-resistant *K. pneumoniae* with rifampicin [17]. Defensins, as a family of cationic AMPs, exert diverse antibacterial and immunomodulatory functions [18]. Multiple β-defensins could coordinate together to enhance antibacterial immune response in airway [19]. Epithelial-cell-derived defensins could trigger neutrophil release of IL-1β and Cxcl2 to restrict *Staphylococcus aureus* infection [20]. Human beta-defensin-3 could increase the survival rate of *K. pneumoniae*-infected mice [21]. Thus, AMPs are expected to be one of the candidate strategies for solving the problem of drug-resistant *K. pneumoniae* infection. Bovine neutrophil β-defensin-5 (B5) was first discovered in neutrophils in 1993 [22], and also expressed in alveolar macrophages, tracheal epithelial cells, and mammary epithelial cells, and mastitis strongly increased B5 expression [23,24]. Our previous studies found that B5 activated macrophages and dendritic cells in vitro, induced effective respiratory mucosal immune response against *Mycobacterium bovis*, and ameliorated *Actinobacillus pleuropneumoniae*-caused pulmonary immunosuppression in vivo [25,26,27]. However, the role of B5 in restricting *K. pneumoniae* infection was unknown.

Innate immune response in lungs is an attractive target for the control of *K. pneumoniae* infection [28,29]. Innate immune effectors, including neutrophils, macrophages, and complement pathways, are critical in the early control of *K. pneumoniae* infection, and neutrophils are the most commonly used effectors [28]. A recent study showed that phagocytosis is a primary determinant of pulmonary clearance of *K. pneumoniae* [30]. Promoting pro-inflammatory cytokine responses contributed to the enhancement of the early host defense against *K. pneumoniae* infection [29]. A vaccine via the intrapulmonary route induced pulmonary protective immunity against *K. pneumoniae* via fibroblast IL-17R signaling [31]. A recent study found that oral *Lacticaseibacillus rhamnosus* CRL1505 could regulate pulmonary innate immune response against *K. pneumoniae* [32]. However, inflammatory response is also the basis for the development of acute lung injury. Most antibiotics can kill bacteria but do not protect from lung damage due to excessive immune response. Chrysophanol [33] and Fei-Yan-Qing-Hua decoction [34] could alleviate *K. pneumoniae*-caused acute lung injury by inhibiting excessive inflammatory response. Therefore, the inhibition of excessive inflammatory response using natural medicine is also a candidate strategy for controlling *K. pneumoniae* infection.

In this study, the effects of B5 on the regulation of the inflammatory response and metabolic response against *K. pneumoniae* infection were investigated. This study showed that B5 could not kill *K. pneumoniae* in vitro; however, intranasal B5 promoted the recruitment of macrophages and dendritic cells in the lungs in the early infection phase, and inhibited the secretion of TNF-α, IL-1β, and IL-17 in the lungs in the later infection phase. Furthermore, B5 suppressed the *K. pneumoniae*-caused excessive activation of signaling pathways associated with NK cell-mediated cytotoxicity, IL-17 signaling pathway, inflammatory response, apoptosis, and necroptosis in the lungs, and regulated glycerophospholipid metabolism and metabolic pathways in the lungs. Importantly, B5 provided protection against *K. pneumoniae* via reducing the bacterial load in the lungs and spleen, and by alleviating the pathological damage of the lungs in mice. Overall, our study first found that the non-bactericidal antibacterial peptide B5 offered protection against multidrug-resistant *K. pneumoniae* by regulating pulmonary innate immune response, inflammatory response, and metabolic response. These findings provide insights into B5 regulating innate immune response and metabolic response in the lungs to restrict multidrug-resistant *K. pneumoniae* invasion.

## 2. Results

### 2.1. B5 Provides Protection against K. pneumoniae

**Figure 1:**
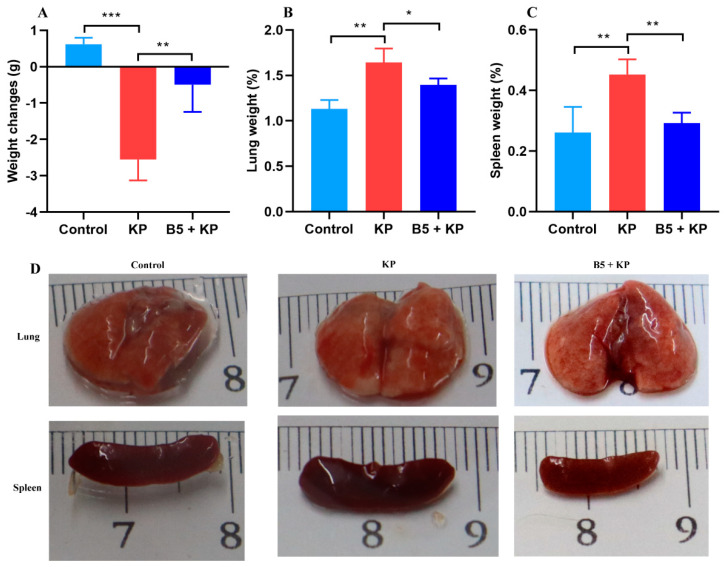
B5 ameliorates *K. pneumoniae*-caused weight loss and organ enlargement. Mice were treated intranasally with B5 (20 μg) and challenged with *K. pneumoniae* three days after treatment and euthanized at 24 h after challenge. (**A**) Body weight changes. (**B**) Weight of lung tissues. (**C**) Weight of spleen tissues. (**D**) Gross pathology of lungs and spleen. Data shown are means ± SD. Data are representative of two independent experiments (*n* = 8 mice per group). Statistical analysis was performed by ANOVA followed by Tukey’s multiple comparison test (* *p* < 0.033, ** *p* < 0.0021, *** *p* < 0.0002).

**Figure 2:**
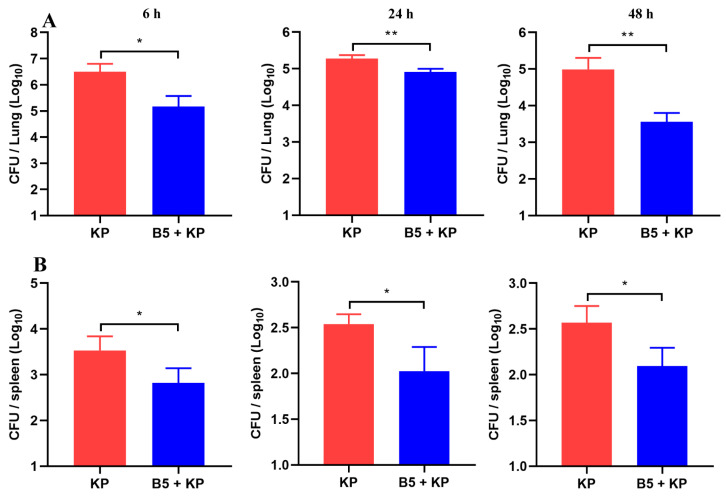
B5 reduces bacterial load in lungs and spleen. Lung and spleen tissues were collected at 6 h, 24 h, and 48 h after challenge. (**A**) Bacterial load in lungs. (**B**) Bacterial load in spleen. Data shown are means ± SD. Data are representative of two independent experiments (*n* = 8 mice per group). Statistical analysis was performed by ANOVA followed by Tukey’s multiple comparison test (* *p* < 0.033, ** *p* < 0.0021).

To determine the bactericidal activity of B5 in vitro, 0–1.28 mg/mL of B5 was incubated with 10^5^ CFU/mL of *K. pneumoniae* suspension for 2 h, 4 h, and 6 h at 37 °C, and results showed that B5 failed to kill *K. pneumoniae* (Supplemental Figure S1). To investigate the protection provided by B5 against *K. pneumoniae* in vivo, B5-pretreated mice were challenged with *K. pneumoniae* and euthanized at 6 h, 24 h and 48 h after challenge. Our results showed that *K. pneumoniae* challenge led to the loss of body weight and the enlargement of the spleen and lungs; however, B5 significantly alleviated *K. pneumoniae*-caused body weight loss and organ enlargement (Figure 1A,D). Consistently, B5 significantly decreased the *K. pneumoniae*-caused increase in the lung and spleen weight (Figure 1B,C). Furthermore, B5 significantly reduced the bacterial load in the lungs and spleen in both the early and later stages of *K. pneumoniae* infection (Figure 2A,B). Collectively, these results suggest that B5 provides protection against *K. pneumoniae*.

### 2.2. B5 Up-Regulates Pulmonary mRNA Expressions of Cytokines and Chemokines in Early Infection Phase

**Figure 3:**
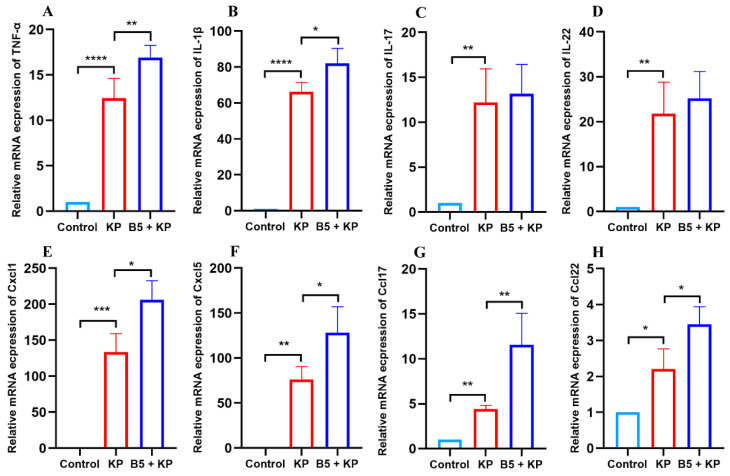
B5 up-regulates pulmonary mRNA expressions of cytokines and chemokines in early infection phase. Lung and spleen tissues were collected at 4 h after challenge. (**A**–**D**) The mRNA expression levels of TNF-α (**A**), IL-1β (**B**), IL-17 (**C**), and IL-22 (**D**) in lungs. (**E**–**H**) The mRNA expression levels of Cxcl1 (**E**), Cxcl5 (**F**), Ccl17 (**G**), and Ccl22 (**H**) in lungs. Data shown are means ± SD. Data are representative of two independent experiments (*n* = 3). Statistical analysis was performed by ANOVA followed by Tukey’s multiple comparison test (* *p* < 0.033, ** *p* < 0.0021, *** *p* < 0.0002, **** *p* < 0.0001).

The inflammatory response is vital to bacterial clearance in the early infection phase. To investigate the effects of B5 on the mRNA expressions of cytokines and chemokines in the early infection phase, the lungs were collected at 4 h after *K. pneumoniae* challenge. Results showed that *K. pneumoniae* challenge significantly increased the mRNA expressions of TNF-α, IL-1β, IL-17, IL-22, Cxcl1, Cxcl5, Ccl17, and Ccl22 in the lungs compared with the uninfected control, and B5 pretreatment significantly increased the mRNA expressions of TNF-α, IL-1β, Cxcl1, Cxcl5, Ccl17, and Ccl22 in the lungs compared with the *K. pneumoniae* infection control (Figure 3A–H). These results suggest that B5 promotes inflammatory response induced by *K. pneumoniae* in the early infection phase.

### 2.3. B5 Increases Pulmonary Macrophages and Dendritic Cells in Early Infection Phase

**Figure 4:**
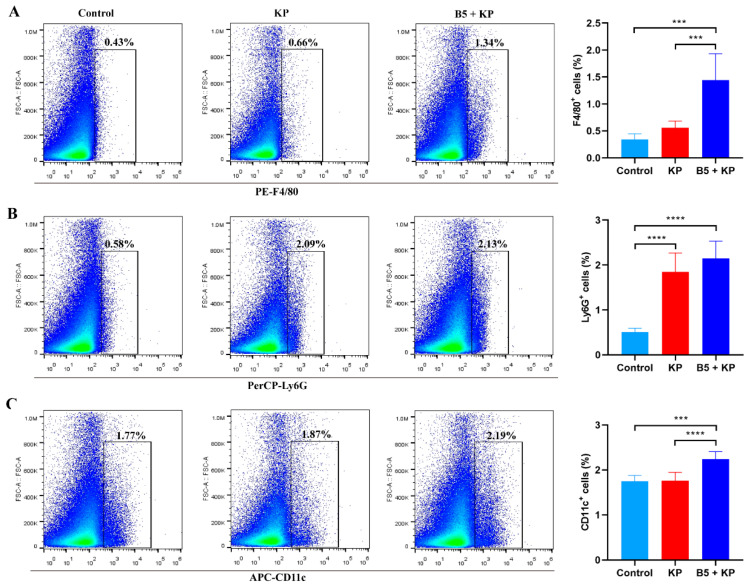
B5 promotes the recruitment of innate immune cells to the lungs in the early infection phase. The lungs were collected at 6 h after challenge. (**A**) Percentage of macrophages (F4/80^+^ cells) in the lungs. (**B**) Percentage of neutrophils (Ly6G^+^ cells) in the lungs. (**C**) Percentage of dendritic cells (CD11c^+^ cells) in the lungs. Data shown are means ± SD. Data are representative of two independent experiments (*n* = 8 mice per group). Statistical analysis was performed by ANOVA followed by Tukey’s multiple comparison test (*** *p* < 0.0002, **** *p* < 0.0001).

The mononuclear phagocyte system plays an important role in the innate immune response, which possesses phagocytic function and coordinates antibacterial immune response. Neutrophils and macrophages are important phagocytes. Neutrophils are the effector cells that are first present at the infection sites. To further investigate the effects of B5 on innate immune cells, neutrophils, macrophages, and dendritic cells in lung tissues were determined at 4 h after *K. pneumoniae* challenge. Results showed that *K. pneumoniae* infection significantly increased the number of neutrophils in the lungs compared with the control group, but did not affect macrophages and dendritic cells, suggesting that neutrophils were the first to reach the site of infection (Figure 4A–C). When compared to the KP group, B5 significantly increased the percentage of macrophages and dendritic cells in the lungs, but there was no significant difference in neutrophils between both groups (Figure 4A–C). Overall, these results reveal that B5 enhances the recruitment of macrophages and dendritic cells into the lungs in the early infection stage.

### 2.4. B5 Regulates Levels of Myeloperoxidase and Lysozyme in Serum in Later Infection Phase

**Figure 5:**
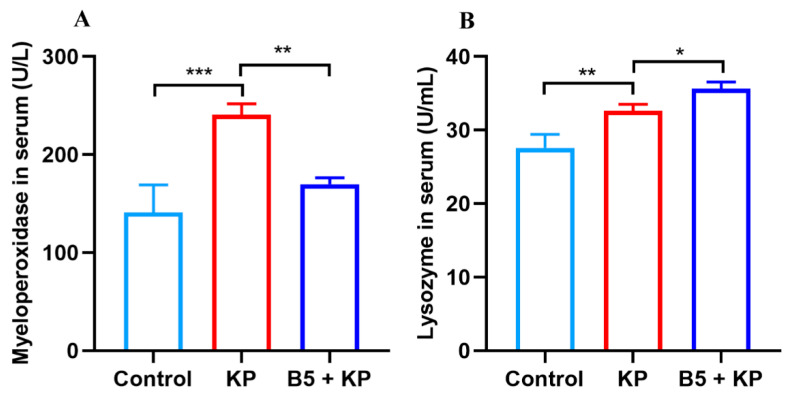
B5 regulates levels of myeloperoxidase and lysozyme in serum in later infection phase. Serum samples were collected at 24 h after challenge. (**A**) The level of myeloperoxidase in serum. (**B**) The level of lysozyme in serum. Data shown are means ± SD. Data are representative of two independent experiments (*n* = 3). Statistical analysis was performed by ANOVA followed by Tukey’s multiple comparison test (* *p* < 0.033, ** *p* < 0.0021, *** *p* < 0.0002).

Myeloperoxidase is a local mediator of tissue damage in various inflammatory diseases. Lysozyme is important for bacterial killing and the resolution of inflammation at mucosal sites. To investigate the effects of B5 on the levels of myeloperoxidase and lysozyme in the later infection phase, serum samples were collected at 24 h after *K. pneumoniae* challenge. Results showed that *K. pneumoniae* infection significantly increased the activity of myeloperoxidase compared with the uninfected control; however, B5 markedly decreased the activity of myeloperoxidase (Figure 5A). Moreover, B5 significantly enhanced the production of lysozyme compared with the untreated control (Figure 5B). Overall, these results reveal that B5 decreases the level of myeloperoxidase and promotes the production of lysozyme in the later infection phase.

### 2.5. B5 Ameliorates K. pneumoniae-Induced Pathological Damage in Lungs in Later Infection Phase

**Figure 6:**
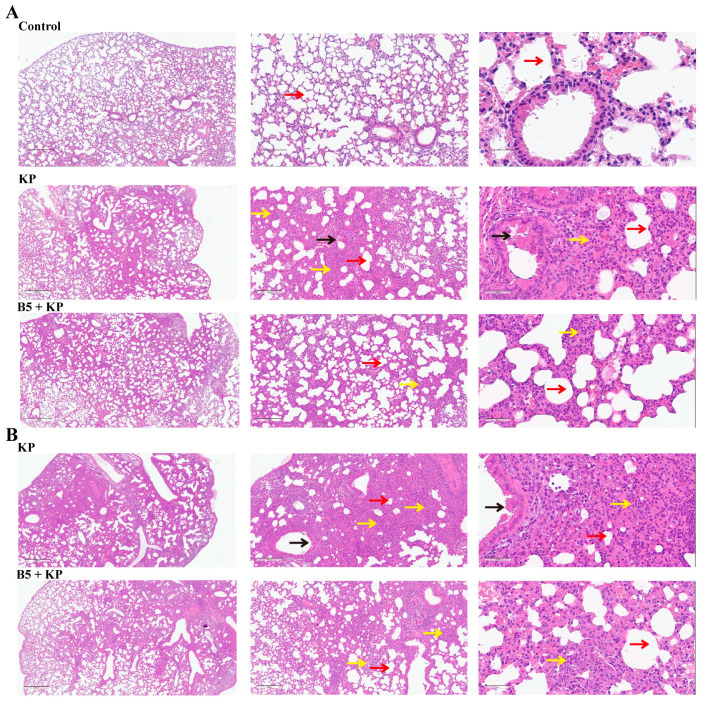
B5 alleviates inflammatory damage in lungs. (**A**) Histopathological images of lungs at 24 h after challenge. (**B**) Histopathological images of lungs at 48 h after challenge. Left images at ×40 magnification (scale bar, 500 μm); middle images at ×100 magnification (scale bar, 200 μm); right images at ×400 magnification (scale bar, 50 μm). Red arrows represent alveoli, black arrows represent fallen bronchial epithelial cells, and yellow arrows represent inflammatory cells in pulmonary interstitium. Data are representative of two independent experiments (*n* = 3 mice per group).

To observe the effects of B5 on *K. pneumoniae*-induced pathological damage in lung tissues, the lungs were collected at 24 h and 48 h after *K. pneumoniae* challenge and stained with HE. As shown in Figure 6, *K. pneumoniae* infection led to a high inflammatory cell infiltration in lung tissues compared with the control group. Moreover, the thickening of alveolar septa, damaged alveolar structure, fallen bronchial epithelial cells, and excessive secretions in the lungs of *K. pneumoniae*-challenged mice were observed. However, B5 pretreatment markedly decreased inflammatory cells and secretions compared with the *K. pneumoniae* control. Notably, as the *K. pneumoniae* infection prolonged, the inflammatory response in the lungs intensified, but B5 pretreatment obviously delayed the development of inflammatory response (Figure 6A,B). Overall, these results suggest that B5 ameliorates *K. pneumoniae*-caused pathological damage in the lungs.

### 2.6. B5 Down-Regulates Pulmonary Cytokine Production in Later Infection Phase

**Figure 7:**
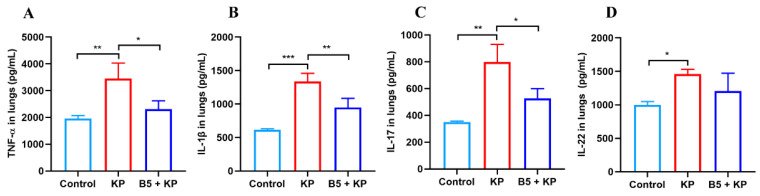
B5 decreases pro-inflammatory cytokine secretion in the lungs in the later stage of *K. pneumoniae* infection. (**A**–**D**) Levels of TNF-α, IL-1β, IL-22, and IL-17 in the lungs at 24 h after challenge. Data shown are means ± SD. Data are representative of two independent experiments (*n* = 3). Statistical analysis was performed by ANOVA followed by Tukey’s multiple comparison test (* *p* < 0.033, ** *p* < 0.0021, *** *p* < 0.0002).

Pro-inflammatory cytokines were crucial for restricting *K. pneumoniae* in the early infection phase, but excessive inflammatory response causes pathological damage [35]. To further investigate the effects of B5 on inflammatory response, cytokines in lung tissues were measured 24 h after *K. pneumoniae* challenge. As shown in Figure 7, *K. pneumoniae* challenge led to a significant increase in the production of TNF-α, IL-1β, IL-22, and IL-17 in the lungs compared with the uninfected control, suggesting that obvious inflammatory response occurred in the lungs, which was in line with the pathological changes in the lungs. In comparison with the *K. pneumoniae* group, B5 pretreatment significantly inhibited the production of TNF-α, IL-1β, and IL-17 and mildly inhibited the secretion of IL-22, but no significant difference in IL-22 between the KP group and the B5 + KP group was observed (Figure 7A–D). These results reveal that B5 suppresses the excessive secretion of TNF-α, IL-1β, and IL-17 in the later infection phase.

### 2.7. B5 Regulates Gene Expression Profile in Lungs

**Figure 8:**
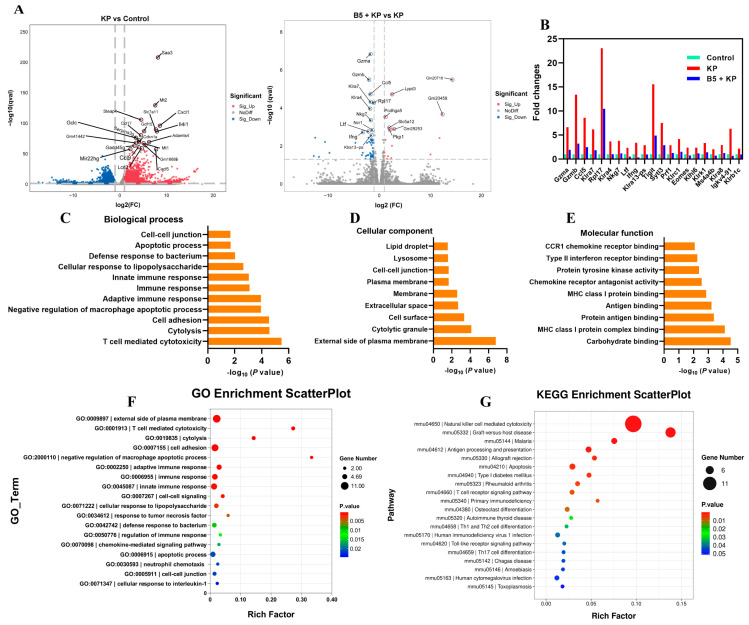
Gene Ontology (GO) term and Kyoto Encyclopedia of Genes and Genomes (KEGG) pathway analysis of differentially expressed genes. (**A**) Differentially expressed genes are shown in volcano plots. (**B**) Fold changes in representative differentially expressed genes. (**C**–**E**) Gene enrichment in biological process (**C**), cellular component (**D**), and molecular function (**E**). (**F**) GO enrichment scatterplot. (**G**) KEGG enrichment scatterplot.

**Figure 9:**
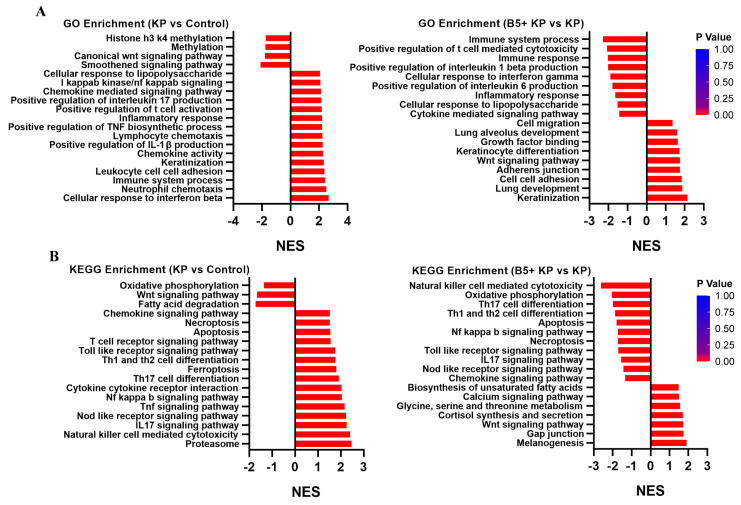
Gene set enrichment analysis (GSEA) of differentially expressed genes. (**A**) GSEA of Gene Ontology (GO) enrichment. (**B**) GSEA of Kyoto Encyclopedia of Genes and Genomes (KEGG) enrichment.

To explore the effects of B5 on the gene expression profiles in the lungs, the lungs were collected at 6 h after challenge, and the RNA-seq experiments were carried out. Results showed that *K. pneumoniae* infection significantly up-regulated 2112 genes (fold change, FC > 2) and significantly down-regulated 3503 genes (FC < 0.5) compared with the uninfected control. Moreover, 16 genes were significantly up-regulated and 54 genes were significantly down-regulated in the B5 + KP group compared with the KP group (Figure 8A). When compared to the control group, *K. pneumoniae* infection induced the overexpression of genes (Gzma, Gzmb, Ccl5, Klra7, Rpl17, etc.) mainly associated with natural killer (NK) cell-mediated cytotoxicity, but B5 significantly inhibited the expression of these genes (Figure 8B), suggesting that B5 may improve the cytotoxicity mediated by the excessive activation of NK cells caused by *K. pneumoniae*. Next, GO analysis showed that in the B5 + KP group and KP group, genes were mainly enriched in T cell-mediated cytotoxicity, defense response to bacterium, immune response, cellular response to lipopolysaccharide, etc. in the biological process, enriched in the external side of plasma membrane, cytolytic granule, cell surface, etc. in the cellular component, and enriched in carbohydrate binding, MHC class I protein complex binding, CCR1 chemokine receptor binding, etc. in the molecular function (Figure 8C–F). Moreover, KEGG analysis showed that genes were mainly enriched in NK cell-mediated cytotoxicity, antigen processing and presentation, Th1 and Th2 cell differentiation, Th17 cell differentiation, Toll-like receptor signaling pathway, and T cell receptor signaling pathway in the B5 + KP group and KP group (Figure 8G). Furthermore, gene set enrichment analysis (GSEA) showed that *K. pneumoniae* infection activated cellular response to interferon beta, immune system process, positive regulation of IL-1β production, inflammatory response, etc. compared with the uninfected control; however, B5 obviously inhibited these responses and enhanced responses associated with lung development, lung alveolus development, growth factor binding, etc. compared with the *K. pneumoniae* control, suggesting that B5 regulated *K. pneumoniae*-induced inflammatory response and enhanced repair response in the lungs (Figure 9A). Consistently, *K. pneumoniae* activated signaling pathways related to NK cell-mediated cytotoxicity, IL-17 signaling pathway, Th17 cell differentiation, apoptosis, necroptosis, and NF-κB signaling pathway in comparison with the uninfected control, but B5 pretreatment inhibited these signaling pathways compared with the *K. pneumoniae* infection control (Figure 9B). Collectively, these results suggest that B5 ameliorates the excessive inflammatory response caused by *K. pneumoniae*.

### 2.8. B5 Regulates Metabolic Response in Lungs

**Figure 10:**
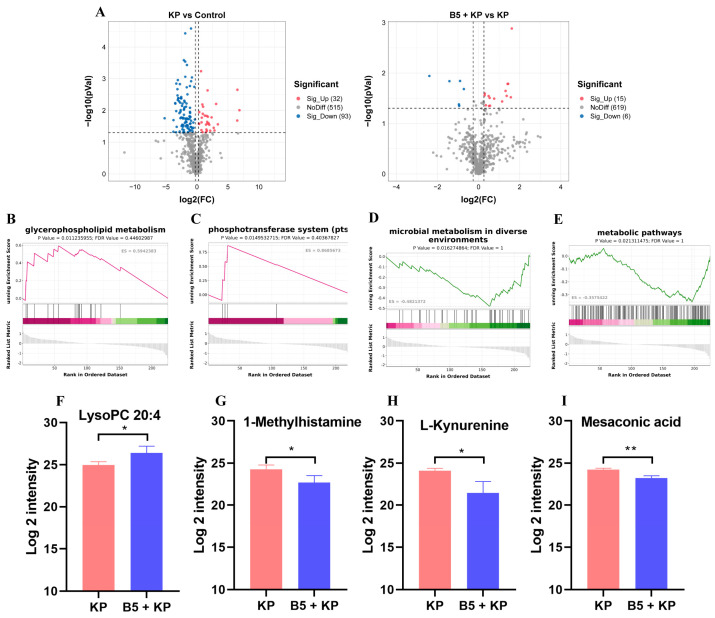
B5 regulates *K. pneumoniae*-induced metabolic response in lungs. (**A**) Differentially expressed metabolites are shown in volcano plots. (**B**–**E**) Gene set enrichment analysis (GSEA) of KEGG enrichment (B5 + KP group vs. KP group). (**F**–**I**) Representative differentially expressed metabolites. Statistical analysis was performed by Student’s *t*-test (* *p* < 0.05, ** *p* < 0.005).

To investigate the effects of B5 on the metabolic response in the lungs, the lungs were collected at 6 h after challenge, and the metabolomic analysis was carried out. Results showed that *K. pneumoniae* infection significantly up-regulated 32 metabolites and down-regulated 93 metabolites compared with the control. Additionally, 15 metabolites were significantly up-regulated and 6 metabolites were significantly down-regulated in the B5 + KP group compared with the KP group (Figure 10A, Supplemental Tables S1 and S2). GSEA showed that *K. pneumoniae* infection led to the suppression of glycerophospholipid metabolism, the phosphotransferase system, the activation of microbial metabolism in diverse environments, and metabolic pathways compared with the uninfected control. However, in comparison with the KP group, B5 significantly reversed these metabolic responses (Figure 10B–E). Moreover, B5 significantly increased LysoPC 20:4 and obviously decreased 1-Methylhistamine, L-Kynurenine, and mesaconic acid compared to the *K. pneumoniae* infection control (Figure 10F–I). Overall, these results suggest that B5 regulates metabolic response in the lungs of mice challenged by *K. pneumoniae*.

## 3. Discussion

Currently, antimicrobial resistance is one of the most serious threats to global public health. Carbapenem-resistant *Enterobacterales*, especially carbapenem-resistant *K. pneumoniae* that poses a major public health problem due to its frequent causative involvement in nosocomial infections (particularly in pneumonia), lead to a high economic burden and mortality rate [36]. Notably, drug-resistant *K. pneumoniae* also exists in pig feed [37] and broiler chicken farms [38], suggesting the close relationships between *K. pneumoniae* from humans and livestock. Therefore, new strategies to prevent or treat *K. pneumoniae* infection are urgently needed.

Antimicrobial peptides are promising candidate drugs to solve the problem of antibiotic resistance, and researchers have conducted clinical trials on AMPs to control drug-resistant bacterial infections [11]. We previously found that the antimicrobial peptide B5 promoted vaccine-induced immune response against *Mycobacterium bovis* [25] and enhanced mucosal defense against *Actinobacillus pleuropneumoniae* [27]. Our previous studies focused on the role of B5 in promoting immune response [25,27]. This study highlighted the roles of B5 in providing protection against multidrug-resistant *K. pneumoniae* by inhibiting excessive inflammatory response and revealed the different or even opposite roles of B5 in alleviating bacterial pneumonia under different pathological conditions. In this study, we provide evidence that intranasal B5 regulates excessive inflammatory response and metabolic response and provides protection against *K. pneumoniae* via reducing the bacterial load in the lungs and spleen, and by alleviating pulmonary pathological damage. The protection is related to the rapid recruitment of macrophages and dendritic cells to the lungs in the early infection phase, the suppression of TNF-α, IL-1β, and IL-17 secretion in the later infection phase, and the inhibition of IL-17 and NF-κB signaling pathways, as well as NK cell-mediated cytotoxicity. We propose that B5 alleviates excessive inflammatory response caused by *K. pneumoniae*, promotes glycerophospholipid metabolism, and inhibits metabolic pathways.

AMPs have direct bactericidal and immunomodulatory capabilities [10,11]. The antimicrobial peptides WAM-1, AA139, and SET-M33 could inhibit *K. pneumoniae* growth and kill *K. pneumoniae* in vitro [14,39]. A recent study showed that levofloxacin and Synoeca-MP peptide from *Synoeca surinama* had synergistic activity and immunomodulatory potential against multidrug-resistant *K. pneumoniae* [40]. The novel antimicrobial peptide K11 exhibited antibacterial and antibiofilm activities without inducing resistance and acted synergistically with antibiotics against drug-resistant *K. pneumoniae* [41]. In the current study, B5 failed to directly kill *K. pneumoniae* in vitro, but it restricted bacterial growth in vivo via regulating inflammatory response and immune response and improved the *K. pneumoniae*-caused weight loss of mice and the enlargement of the lungs and spleen. These findings were similar to an early study [42], and the antimicrobial peptide IDR-1018 provided protection against *Mycobacterium tuberculosis* via regulating innate immune response, enhancing immune cell recruitment, and suppressing harmful inflammatory responses [42]. Moreover, although we did not compare the differences in the secondary structure of B5 derived from recombinant *Pichia pastoris* and chemically synthesized B5, they both promoted the secretion of TNF-α and IL-1β in macrophages and enhanced pulmonary mucosal immunity against bacterial infections [25,26,27], and we supposed that B5-induced protection was non-specific, and B5 may be used as an adjuvant to the existing antimicrobial therapy to control multiple bacterial infections in the respiratory tract.

TNF-α and IL-1β play an important role in the clearance of bacteria and viruses during early infection, but their overproduction could trigger an inflammatory storm, which damages tissues and organs [43]. IL-17 is critical for host defense against extracellular bacterial and fungal pathogens. IL-17R signaling plays a critical role in regulating Cxcl5, which is essential for mucosal immunity against *K. pneumoniae* [44]. In the current study, we found that B5 promoted the mRNA expression of TNF-α and IL-1β in the early infection phase, but inhibited the secretion of TNF-α, IL-1β, and IL-17 in the later infection phase, suggesting that B5 could regulate inflammatory response induced by *K. pneumoniae*. But B5 had no significant effect on the production of IL-22, which was different from our previous study [27], and this may be related to the difference in pathogenic bacteria; moreover, *K. pneumoniae* promoted IL-22 secretion, while APP inhibited IL-22 production in the lungs at 6 h after challenge, and B5 exhibited different immunomodulatory activities in different bacterial pneumonia. In line with the changes in the cytokine levels, B5 visibly alleviated the *K. pneumoniae*-induced infiltration of massive inflammatory cells and the structural damage of bronchial and alveolar cells in the lungs. Our study supported that B5 provided protection against *K. pneumoniae* by inhibiting excessive inflammatory response. In our previous study, we found that B5 activated immune response in the lungs of *Actinobacillus pleuropneumoniae*-infected mice in the early stage of infection [27]. This difference may be due to the pulmonary inflammatory damage caused by *K. pneumoniae* at 6 h after challenge, while APP caused pulmonary immune suppression. We speculated that B5 played different roles in different pathological environments, which was consistent with the regulatory features of AMPs with immunomodulatory functions including the suppression of inflammation, immune activation, etc. [10]. For example, on the one hand, LL-37 could activate the innate immune response to facilitate the host defense against viruses [45]. On the other hand, LL-37 ameliorated methicillin-resistant *staphylococcus aureus*-induced pneumonia by attenuating pro-inflammatory cytokine release [46] and alleviated sepsis via the suppression of the pro-inflammatory macrophage pyroptosis [47]. Myeloperoxidase plays an important role in killing *K. pneumoniae* and inactivating neutrophil elastase to enhance host defense [48], but the excessive production of myeloperoxidase exacerbates lung inflammation [49]. Lysozyme can kill bacteria and modulate the host immune response [50]. In this study, we found that B5 decreased the *K. pneumoniae*-induced high levels of myeloperoxidase and enhanced the production of lysozymes in serum, suggesting the effective activity of B5 in regulating inflammatory response and enhancing bacterial killing.

Innate immune cells, including macrophages, neutrophils, and dendritic cells, play a critical role in bacterial clearance and regulating innate immune response via the production of cytokines and chemokines in the early phase of bacterial infection [51]. When *K. pneumoniae* establishes infection, neutrophils are the effector cells that first reach the sites of bacterial infection through chemokines and cytokines produced by macrophages, and they have greater phagocytic and killing capacities than alveolar macrophages and are vital to the containment and clearance of *K. pneumoniae*. Alveolar macrophages help control *K. pneumoniae* infection mainly through recruiting neutrophils [51]. Antimicrobial peptide IDR-1018 helps to destroy *Mycobacterium tuberculosis* via recruiting defense cells to the infection sites [42]. In line with this, we found that B5 enhanced the recruitment of macrophages and dendritic cells to the lungs in the early stage of *K. pneumoniae* infection, and this was consistent with the bacterial load in the lungs, suggesting that B5 could eliminate pulmonary bacteria via quickly recruiting innate immune cells. Pro-inflammatory chemokines involved in multiple immune functions such as neutrophilic inflammation (e.g., Cxcl1 and Cxcl5) and macrophage as well as Th2 cell responses (e.g., Ccl17 and Ccl22) [52]. In the current study, we found that B5 significantly up-regulated mRNA expression of Cxcl1, Cxcl5, Ccl17, and Ccl22 in the lungs in the early phase of *K. pneumoniae* infection, and this may be a mechanism of B5 for eliciting the recruitment of macrophages and dendritic cells. Additionally, although we did not detect innate immune cells of the lungs in the late phase of infection, histopathological and cytokine testing results showed that B5 obviously alleviated pathological damage of lung tissues both at 24 h and 48 h after challenge and significantly reduced the production of pro-inflammatory cytokines at 24 h after challenge, suggesting that B5 can protect *K. pneumoniae*-challenged mice in the late phase of infection rather than by inducing excessive immune cell activity.

The innate immune response is classified as the first line of defense against pathogens. However, lung infection with *K. pneumoniae* caused the infiltration of massive inflammatory cells, resulting in the disruption of the mechanical barrier of the respiratory tract and the disturbance of the host defense [53]. In our study, RNA-seq results showed that the inflammatory response, necroptosis, apoptosis, and NF-κB signaling pathway in the lungs were triggered at 6 h after *K. pneumoniae* challenge, suggesting that inflammatory injury occurred, and these findings were in line with the cytokine levels in the lungs. However, B5 mitigated these responses, suggesting that B5 protected *K. pneumoniae*-challenged mice via inhibiting inflammatory response and cell death, which was consistent with histopathological changes in the lungs. Moreover, granzymes (Gzma and Gzmb) mainly expressed by NK cells regulated the local inflammatory response during *K. pneumoniae*-caused pneumonia [54]. Quick and effective NK-mediated immune responses are vital to eliminating pathogens and promoting adaptive response in the lung. However, the immunopathology exacerbated by excessive NK cell responses leads to the acute death of the host during influenza virus infection [55]. In the current study, we found that *K. pneumoniae* significantly activates NK cell-mediated cytotoxicity in the early phase of infection, but B5 reversed this change. However, further study will be needed to clarify effects of B5 on NK cell activity.

A recent study reported that *K. pneumoniae* activates host glutaminolysis and fatty acid oxidation to induce host metabolic stress, which facilitates tolerance to pulmonary infection [56]. Glycerophospholipid metabolism participates in inflammatory response. The regulation of glycerophospholipid metabolism using natural medicines could alleviate acute ulcerative colitis [57] and lipopolysaccharide-induced sepsis [58]. In this study, *K. pneumoniae* infection caused the suppression of glycerophospholipid metabolism, the phosphotransferase system, the activation of microbial metabolism in diverse environments, and metabolic pathways, while B5 pretreatment significantly reversed these metabolic responses. Moreover, B5 significantly decreased the production of 1-Methylhistamine, plasmenyl-PE 37:4, L-Kynurenine, and mesaconic acid compared to the *K. pneumoniae* infection control. However, further study will be needed to identify and quantify these metabolites.

**Figure 11:**
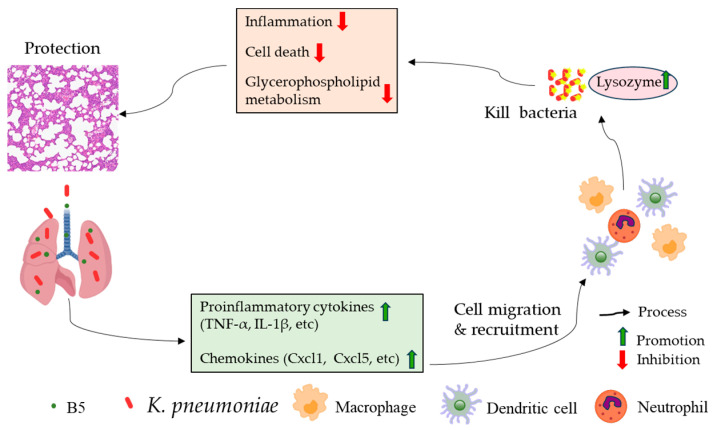
The mechanism and mode of action of B5 against *K. pneumoniae* infection.

In summary, B5 promoted the rapid recruitment of pulmonary macrophages and dendritic cells in the early stage of *K. pneumoniae* infection, down-regulated the levels of TNF-α, IL-1β, and IL-17 in the lungs in the later stage of *K. pneumoniae* infection, and inhibited the activation of signaling pathways associated with inflammatory response, apoptosis, necrosis, etc. (Figure 11). Importantly, B5 significantly decreased the bacterial load in the lungs and spleen and visibly alleviated pulmonary histopathological damage (Figure 11). Collectively, B5 effectively provided protection against multidrug-resistant *K. pneumoniae* via ameliorating the excessive inflammatory response. Nonetheless, the mechanism of B5 in regulating inflammatory response and metabolic response needs to be clarified. This study provides a possibility that B5 can be used as an alternative adjuvant drug promoting multidrug-resistant *K. pneumoniae* clearance and mitigating pulmonary inflammatory damage caused by *K. pneumoniae*.

## 4. Materials and Methods

### 4.1. Mice

The protocols and procedures of mice experiments were performed according to the protocols for the care of laboratory animals, Ministry of Science and Technology, People’s Republic of China, and approved according to animal care and use committee protocols with license number of GXU20230163 at the Guangxi University, Nanning, China. The C57BL/6 mice were purchased from SiPeiFu Biotechnology Co., Ltd. (Beijing, China) and were kept under specific pathogen-free conditions. Mice received access to food and water ad libitum.

### 4.2. Reagents

Tumor necrosis factor (TNF)-α, interleukin (IL)-1β, IL-17, and IL-22 ELISA kits were purchased from Neobioscience (Shenzhen, China). Myeloperoxidase and lysozyme detection commercial kits were purchased from Nanjing Jiancheng Bioengineering Institute (Nanjing, Chian). RNAiso Plus, the first-strand cDNA synthesis kit, and the SYBR Green system were purchased from SparkJade (Qingdao, China). Purified anti-mouse CD16/32, FITC-CD11c, PE-F4/80, and PerCP-Ly6G were purchased from Elabscience (Wuhan, China). Other reagents were from Solarbio. B5 (amino acid sequence: NPQSCRWNMGVCIPISCPGNMRQIGTCFGPRVPCCRRW) was synthesized at Shanghai Apeptide Co., Ltd. (Shanghai, China).

### 4.3. Bacterial Culture

The *K. pneumoniae* strain was kept by the Pharmacology and Pathology Laboratory of School of Animal Science and Technology, Guangxi University. The strain was resistant to 12 antibiotics including vancomycin, tetracycline, ampicillin, polymyxin, lincomycin, and so on. *K. pneumoniae* was grown in Luria–Bertani (LB) liquid medium, and 100 μL of the bacterial suspension was added to 10 mL of LB liquid medium and cultured in bacterial culture shaker at 160 rpm for 16–24 h at 37 °C. Bacterial suspension was diluted with PBS, and serial 10-fold dilutions were plated on LB solid medium.

### 4.4. B5 Pretreatment and K. pneumoniae-Induced Pneumonia in Mice

Female C57BL/6 mice, six to eight weeks old, were randomly divided into three groups: (1) control group (PBS control); (2) KP group (*K. pneumoniae* challenge control); and (3) B5 + KP group (*K. pneumoniae* challenge after B5 pretreatment). Mice were pretreated intranasally with 20 μg of B5 three times at two-day intervals. The dose of B5 was chosen according to our previous study [25]. Negative and infection control mice received equal volumes of PBS. Mice were challenged with 10^8^ CFU of *K. pneumoniae* via the intranasal route three days after pretreatment. Next, mice were euthanized at 4 h, 6 h, 24 h, and 48 h after challenge to collect samples for subsequent measurement. According to a previous study [12], 6 h before infection was classified as the early infection phase and 24 h after infection was classified as the mid-to-late infection phase.

### 4.5. Bacterial Load Assay

Bacterial load assay was performed by plating serial dilutions of homogenized lung and spleen tissues on MaConkey agar medium to quantify colonies of *K. pneumoniae*. Tissues were homogenized in 1 mL of PBS using a tissue homogenizer apparatus (Servicebio, Wuhan, China). Tissue homogenates were diluted with PBS, and serial 10-fold dilutions were plated on MaConkey agar solid plates. Then, 100 μL of samples from each dilution were inoculated on plates in triplicate and cultured for 18–24 h at 37 °C. Colonies of *K. pneumoniae* in each culture plate were counted.

### 4.6. Histopathological Assessments

Morphological and histopathological analyses were performed to assess the protective effects of B5 pretreatment in *K. pneumoniae*-challenged mice. Lungs were collected at 24 h and 48 h after *K. pneumoniae* challenge, fixed in 10% formaldehyde, embedded in paraffin, cut into 3 μm sections, and stained with hematoxylin and eosin (HE) to observe the histopathological changes in lung tissues.

### 4.7. ELISA

Lung tissues were collected 24 h after *K. pneumoniae* challenge, and the concentrations of TNF-α, IL-1β, IL-17, and IL-22 in the supernatants of homogenized equivalent lung tissues (1:100 dilution) using cold PBS were measured using mouse ELISA kits according to the manufacturer’s instructions. Serum samples were collected 24 h after *K. pneumoniae* challenge, and the levels of myeloperoxidase and lysozyme in serum were detected using commercial kits according to the manufacturer’s instructions. The optical density (OD) values were quantified via measuring the absorbance at 450 nm wavelength using ELISA plate reader (Tecan (Shanghai) Trading Co., Ltd., Shanghai, China, Infinite M200 Pro, Zürich, Switzerland).

### 4.8. RT-qPCR

Lung tissues were harvested 4 h after *K. pneumoniae* challenge, and the total RNA was isolated using RNAiso Plus. Reverse transcription was performed using the first-strand cDNA synthesis kit. Real-time PCR was performed on the cDNA samples using the SYBR Green system. The primers used are shown in Table 1. The cycling conditions were as follows: initial denaturation at 95 °C for 30 s, followed by 40 cycles of reaction (95 °C for 5 s, 58–60 °C for 30–60 s, and 72 °C for 60 s). The gene-specific threshold cycles (Ct) of the respective samples were internally normalized using the average Ct value of GAPDH. The ^Δ^Ct values for all experimental samples were subtracted from the ^Δ^Ct values of the control samples (^ΔΔ^Ct). The fold change in the levels of test gene mRNAs was expressed as 2^−ΔΔCt^.

### 4.9. Flow Cytometry

The lung cells were isolated according to our previous study [20]. Briefly, lungs were harvested at 6 h after *K. pneumoniae* challenge and were placed into 20 mm dishes and then digested with collagenase I (1 mg/mL) and Dnase I (30 U/mL) for 30 min at 37 °C. The lung cells were discharged onto a 70 μm filter and incubated with RBC Lysing Buffer to deplete red blood cells. The collected cells were incubated with CD16/32, PE-F4/80, APC-CD11c, and PerCP-Ly6G for 30 min at 4 °C. The stained cells were washed with staining buffer and then fixed with 4% paraformaldehyde. Finally, cells were analyzed using a FACSVerse flow cytometer (Invitrogen, Carlsbad, CA, USA) and FlowJo software version 10.

### 4.10. RNA-Seq

The RNA-seq (RNA sequencing) of the lungs was performed according to our previous study [27]. Briefly, lung tissues were collected at 6 h after *K. pneumoniae* challenge, and the mRNA was purified from the extracted total RNA of the lungs and fragmented into short fragments that were reverse-transcribed to create the cDNA. An A-base was added to the blunt ends of each strand. Dual-index adapters were ligated to the fragments. The U-labeled second-stranded DNAs were treated with heat-labile UDG enzyme (NEB, cat.m0280, Ipswich, MA, USA), and the ligated products were amplified with PCR. The average insert size for the final cDNA libraries were 300 ± 50 bp. Finally, the 2 × 150 bp paired-end sequencing (PE150) was performed on an Illumina Novaseq™ 6000 (LC-Bio Technology CO., Ltd., Hangzhou, China) according to the vendor’s recommended protocol. The differentially expressed genes of RNA-seq were assessed by the DESeq2 software version 3.10.2. The genes with the parameter of false discovery rate below 0.05 and absolute fold change ≥ 2 were considered differentially expressed genes, which were then subjected to enrichment analysis of Gene Ontology (GO) functions and Kyoto Encyclopedia of Genes and Genomes (KEGG) pathways. The screening threshold was set to *p* < 0.05, and when the Pearson correlation coefficient reached *p* < 0.05, it was considered to be statistically significant.

### 4.11. Metabolomic Analysis

Lung tissues were collected at 6 h after *K. pneumoniae* challenge. Metabolites were extracted from 20 μL of each lung tissue using precooled 50% methanol buffer (120 μL). Then, the mixture of metabolites was incubated for 10 min at room temperature and stored at −20 °C overnight. The supernatant was transferred to 96-well plates after centrifuging at 4000× *g* for 20 min and stored at −80 °C for further LC-MS analysis. Pooled quality control (QC) samples were also prepared by combining 10 uL of each extraction mixture. All samples were analyzed using a TripleTOF 5600 Plus high-resolution tandem mass spectrometer (SCIEX, Warrington, UK) with both positive- and negative-ion modes. Chromatographic separation was performed using a performance liquid chromatography (UPLC) system (SCIEX, UK). An ACQUITY UPLC T3 column (100 mm × 2.1 mm, 1.8 μm, Waters, Wilmslow, UK) was used for the reversed-phase separation. The TripleTOF 5600 Plus system was used to detect metabolites eluted from the column. For the positive-ion mode, the ion spray floating voltage was set at 5 kV, and for the negative-ion mode, it was set at −4.5 kV. The acquired LC-MS data pretreatment was performed using the XCMS software version 3.8.2. The open access databases, KEGG and HMDB, were used to annotate the metabolites by matching the exact molecular mass data (*m*/*z*) to those from the database within a threshold of 10 ppm. PCA was performed to detect outliers and batch effects using the pre-processed dataset. Data normalization was performed on all samples using the probabilistic quotient normalization algorithm. Then, QC-robust spline batch correction was performed using QC samples. The *p* value was analyzed by Student’s *t*-tests, which was then adjusted for multiple tests using an FDR (Benjamini–Hochberg) that was used for the different metabolite selection. The R package “Cluster Profiler” was used to perform gene set enrichment analysis (GSEA). The value of Variable Importance in Projection (VIP) is an indicator used to assess the relationship between a metabolite and sample classification. The VIP > 1 and *p* < 0.05 were set to select distinct metabolites.

### 4.12. Statistical Analysis

All data are expressed as mean ± standard deviation (SD). For comparison between two groups, Student’s *t*-test was applied, and for comparisons among more than two groups, one-way ANOVA followed by Tukey’s multiple comparison test using GraphPad Prism version 8.0 software were performed. Flow cytometry results were analyzed using the FlowJo software version 10. *p* < 0.05 was considered statistically significant.

## Figures and Tables

**Table 1:** Primer sets for the reverse transcriptase-polymerase chain reaction analysis.

Targets	Forward	Reverse
TNF-α	CTCATGCACCACCATCAAGG	ACCTGACCACTCTCCCTTTG
IL-1β	GAAATGCCACCTTTTGACAGTG	TGGATGCTCTCATCAGGACAG
IL-17	TTTAACTCCCTTGGCGCAAAA	CTTTCCCTCCGCATTGACAC
IL-22	ATGAGTTTTTCCCTTATGGGGAC	GCTGGAAGTTGGACACCTCAA
Cxcl1	CTGGGATTCACCTCAAGAACATC	CAGGGTCAAGGCAAGCCTC
Cxcl5	GTTCCATCTCGCCATTCATGC	GCGGCTATGACTGAGGAAGG
Ccl17	TACCATGAGGTCACTTCAGATGC	GCACTCTCGGCCTACATTGG
Ccl22	CTTGCTGTGGCAATTCAGACC	ACTAAACGTGATGGCAGAGGG
GAPDH	GGTGAAGGTCGGTGTGAACGGA	TGTTAGTGGGGTCTCGCTCCTG

## Data Availability

The data that support the findings of this study are available from the corresponding author upon reasonable request (liangzm@cau.edu.cn).

## Supplementary Materials

The following supporting information can be downloaded at https://www.mdpi.com/article/10.3390/ijms251910506/s1.
