# Supplementary material for: Bovine Neutrophil β-Defensin-5 Provides Protection against Multidrug-Resistant Klebsiella pneumoniae via Regulating Pulmonary Inflammatory Response and Metabolic Response
Source: Int J Mol Sci. 2024 Sep 29;25(19):10506. doi: 10.3390/ijms251910506 (PMC11477005; doi:10.3390/ijms251910506)
Supplement: Supplementary file 1 [file ijms-25-10506-s001.zip › ijms-3190162-supplementary.pdf]

Supplementary Materials for

Bovine neutrophil β-defensin-5 provides protection against multidrug-resistant *Klebsiella pneumoniae* via regulating pulmonary inflammatory response and metabolic response

Shuxin Zhu[1,2,3,†], Dejia Dai[1,2,3,†], Han Li[1,2,3], Jingsheng Huang[1,2,3], Weichao Kang[1,2,3], Yunmei Yang[1,2,3], Yawen Zhong[1,2,3], Yifei Xiang[1,2,3], Chengzhi Liu[1,2,3], Jiakang He[1,2,3,*] and Zhengmin Liang[1,2,3,*]

[1] College of Animal Science and Technology, Guangxi University, Nanning 530004, China;

[2] Guangxi Zhuang Autonomous Region Engineering Research Center of Veterinary Biologics, Nanning, 530004, China;

[3] Guangxi Key Laboratory of Animal Reproduction, Breeding and Disease Control, Nanning, 530004, PR China.

[†] These authors contributed equally to this work

[*]Corresponding author: Jiakang He, E-mail: jkhe@gxu.edu.cn

Zhengmin Liang, E-mail: liangzm@gxu.edu.cn

Mailing address: Guangxi University, No. 100 University West Road, 530004, Nanning, China

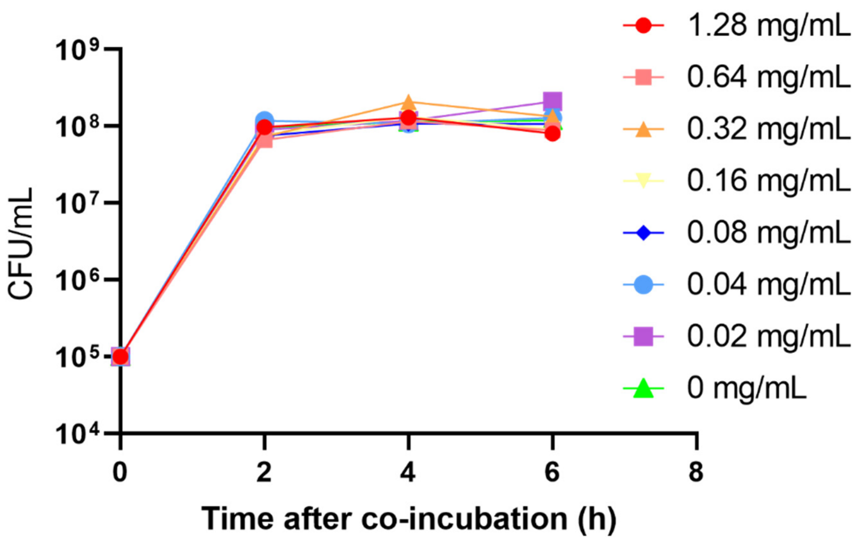

Supplemental Figure S1. The bactericidal activity of B5 against *K. pneumoniae in vitro*. *K. pneumoniae* (10^5 CFU/mL) was incubated with different concentrations of B5 for 2 h, 4 h, and 6 h at 37°C, then bacterial suspension was diluted with PBS and serial 10-fold dilutions were plated on LB solid medium.

Table S1. Differentially expressed metabolites (KP vs Control)

MS2 metabolites name	Fold change	Regulation	p value
PG 31:0; PG(15:0/16:0)	0.03	down	0.02
Thymol Sulfate	0.07	down	0.05
PG 32:0; PG(16:0/16:0)	0.07	down	0.02
PG 30:0; PG(14:0/16:0)	0.09	down	0.01
sn-Glycero-3-phosphocholine	0.09	down	0.01
PG 40:6; PG(18:0/22:6)	0.10	down	0.05
cis-5,8,11,14,17-Eicosapentaenoic acid	0.10	down	0.01
PG 31:1; PG(16:0/15:1)	0.10	down	0.00
Trilobinone	0.10	down	0.03
1-Stearoyl-2-docosahexaenoyl-sn-glycero-3-phospho-(1'-sn-glycerol)	0.11	down	0.00
PG 38:6; PG(16:0/22:6)	0.11	down	0.00
PG 38:5; PG(16:0/22:5)	0.12	down	0.00
PG 42:11; PG(20:5/22:6)	0.12	down	0.00
PG 36:5; PG(16:0/20:5)	0.12	down	0.00
PG 44:12; PG(22:6/22:6)	0.12	down	0.00
20a-Dihydroprogesterone	0.14	down	0.01
LysoPC 20:5	0.14	down	0.01
O,O-Diethyl phosphate	0.14	down	0.05

PG 44:11; PG(22:5/22:6)	0.15	down	0.00
PG 32:1; PG(16:0/16:1)	0.15	down	0.01
LysoPG 22:5; LysoPG 22:5	0.16	down	0.02
PG 30:1; PG(14:0/16:1)	0.16	down	0.01
1-Palmitoyl-2-oleoyl-phosphatidylglycerol	0.16	down	0.01
PG 38:7; PG(16:1/22:6)	0.17	down	0.00
LysoPE 20:5	0.17	down	0.00
PG 33:1; PG(16:0/17:1)	0.17	down	0.02
LysoPC 22:5	0.18	down	0.00
Dodeca-2(E),4(E)-dienoic acid	0.18	down	0.03
6,10,14-Trimethyl-5,9,13-pentadecatrien-2-one	0.19	down	0.01
PI 40:5; PI(20:1/20:4)	0.19	down	0.03
LysoPG 20:5; LysoPG 20:5	0.20	down	0.04
PG 34:2; PG(16:0/18:2)	0.20	down	0.01
Quinceoxepine	0.21	down	0.03
PI 38:6; PI(16:0/22:6)	0.21	down	0.02
4-Pyridoxic acid	0.21	down	0.02
1-Stearoyl-2-arachidonoyl-sn-glycero-3-phospho-(1'-sn-glycerol)	0.21	down	0.05
19-Hydroxy-PGE2	0.22	down	0.03
PI 38:4; PI(18:0/20:4)	0.23	down	0.04

PG 32:3; PG(16:0/16:3)	0.23	down	0.00
.delta.-Dodecalactone	0.23	down	0.01
PG 32:2; PG(16:1/16:1)	0.23	down	0.01
1-(1-Methylethenyl)-4-(1-methylethyl)benzene	0.24	down	0.05
PG 40:7; PG(18:1/22:6)	0.24	down	0.01
PG 36:4; PG(16:0/20:4)	0.24	down	0.00
PG 34:3; PG(16:1/18:2)	0.25	down	0.01
PG 38:7; PG(18:2/20:5)	0.25	down	0.01
13-cis-Retinal	0.26	down	0.02
Prostaglandin I3	0.26	down	0.02
5'-S-Methylthioadenosine	0.27	down	0.00
5'-Methylthioadenosine	0.27	down	0.00
LysoPE 22:5	0.27	down	0.00
LysoPC 14:1	0.28	down	0.03
6-Hydroxynicotinic acid	0.29	down	0.02
Monobutyl phthalate	0.30	down	0.01
anhydroretinol	0.30	down	0.02
PG 44:10; PG(22:4/22:6)	0.31	down	0.02
(-)-Riboflavin	0.31	down	0.01
LysoPG 18:0; LysoPG 18:0	0.32	down	0.03

LysoPC 18:0	0.32	down	0.00
DL-Dopa	0.33	down	0.02
Pelargonic acid	0.33	down	0.00
1-Heptadecanoyl-sn-glycero-3-phosphocholine	0.35	down	0.00
4-Methylhippuric acid	0.35	down	0.04
PG 34:4; PG(14:0/20:4)	0.35	down	0.03
PG 40:6; PG(18:1/22:5)	0.35	down	0.03
PG 42:8; PG(20:4/22:4)	0.36	down	0.02
2-trans-6-cis-Dodecadienal	0.37	down	0.05
3-Methyldioxyindole	0.40	down	0.04
PG 42:9; PG(20:3/22:6)	0.43	down	0.02
PG 42:10; PG(20:4/22:6)	0.43	down	0.02
LysoPC 20:4	0.45	down	0.04
Nonyl acetate	0.46	down	0.00
Heptanoic acid	0.46	down	0.03
3,4-Dihydroxybenzylamine	0.46	down	0.01
S-Adenosyl-L-methionine	0.46	down	0.01
Acylcarnitine 4:0	0.46	down	0.04
Uridine	0.47	down	0.01
3-Methylpentanoic acid	0.47	down	0.02

LysoPC 17:1	0.48	down	0.04
PG 40:8; PG(18:2/22:6)	0.48	down	0.02
LysoPC 14:0	0.49	down	0.03
Inosine	0.49	down	0.03
Adenine	0.52	down	0.00
Caprylic acid	0.52	down	0.02
Azelaic acid	0.52	down	0.00
Acylcarnitine 5:0	0.54	down	0.03
Capric acid	0.57	down	0.00
LysoPC 15:0	0.59	down	0.04
LysoPC 18:2	0.62	down	0.05
Acrylic acid	0.67	down	0.02
2,3,4,5-Tetrahydroxypentanoic acid	0.69	down	0.04
Undecanedioic acid	0.75	down	0.00
Suberic acid	0.80	down	0.02
Palmitic acid	1.25	up	0.03
2-Naphthalenesulfonic acid	1.28	up	0.03
Oxalic acid	1.29	up	0.01
D-2-Phosphoglyceric acid	1.59	up	0.00
Octadecadienoate	1.65	up	0.04
2,3-Diaminosalicylic acid	1.68	up	0.02

Enol-phenylpyruvate	1.72	up	0.02
alpha-Teresantalic acid	1.83	up	0.01
Pristanic acid	1.89	up	0.05
Alpha-D-Glucose	1.92	up	0.01
Mesaconic acid	1.99	up	0.01
DL-.beta.-Hydroxypalmitic acid	2.41	up	0.01
6-Formylumbelliferone	2.76	up	0.03
5-Hydroxy-6E,8Z,11Z,14Z-eicosatetraenoic acid, 1,5-lactone	2.78	up	0.05
2,6-Dihydroxybenzoic acid	2.89	up	0.04
1-(1Z-Octadecenyl)-sn-glycero-3-phosphocholine	2.96	up	0.01
3-Methyl-2-oxovaleric acid	3.04	up	0.04
D-Gluconic acid	3.28	up	0.03
Methyl acrylate	3.29	up	0.00
Phenol sulphate	3.40	up	0.02
3-Oxotetradecanoic acid	3.72	up	0.03
Ergothioneine	3.99	up	0.03
1-Methylhistamine	4.42	up	0.04
p-Cresol sulfate	5.16	up	0.02
L-Kynurenine	6.55	up	0.04
Inosinic acid	7.14	up	0.04

Triamterene	8.55	up	0.01
11-Methoxynoryangonin	10.39	up	0.03
5'-Methoxy-O-desmethylangolensin	91.05	up	0.02
Yangonin	92.50	up	0.00
3'-Hydroxy-3,4,5,4'-tetramethoxystilbene	116.44	up	0.01

Table S2. Differentially expressed metabolites (B5+KP vs KP)

MS2 metabolites name	Fold change	regulation	p value
p-Cresol sulfate	0.19	down	0.01
L-Kynurenine	0.37	down	0.01
Plasmenyl-PE 37:4; PE(P-15:0/22:4)	0.52	down	0.04
1-Methylhistamine	0.52	down	0.04
Methyl acrylate	0.54	down	0.01
Mesaconic acid	0.61	down	0.02
(S)-9-Hydroxy-10-undecenoic acid	1.23	up	0.03
D-2-Phosphoglyceric acid	1.24	up	0.03
Suberic acid	1.28	up	0.04
Undecanedioic acid	1.40	up	0.03
9-Pentadecenoic acid	1.42	up	0.05
Azelaic acid	1.45	up	0.03

Tridecanoic acid	1.47	up	0.04
LysoPC 20:4	1.69	up	0.03
Phenol sulphate	2.22	up	0.04
2-trans-6-cis-Dodecadienal	2.49	up	0.02
Memantine	2.58	up	0.03
N-Undecanoylglycine	2.67	up	0.02
N-Lauroylsarcosine	2.72	up	0.02
N-Decanoylglycine	2.97	up	0.03
Antazoline	3.07	up	0.00
